# Systematic comparison of 2A peptides for cloning multi-genes in a polycistronic vector

**DOI:** 10.1038/s41598-017-02460-2

**Published:** 2017-05-19

**Authors:** Ziqing Liu, Olivia Chen, J. Blake Joseph Wall, Michael Zheng, Yang Zhou, Li Wang, Haley Ruth Vaseghi, Li Qian, Jiandong Liu

**Affiliations:** 1 Department of Pathology and Laboratory Medicine, University of North Carolina, Chapel Hill, NC 27599 USA; 2 McAllister Heart Institute, University of North Carolina, Chapel Hill, NC 27599 USA; 3 Lineberger Comprehensive Cancer Center, University of North Carolina, Chapel Hill, NC 27599 USA

## Abstract

Cloning of multiple genes in a single vector has greatly facilitated both basic and translational studies that require co-expression of multiple factors or multi-units of complex protein. Many strategies have been adopted, among which 2A “self-cleaving” peptides have garnered increased interest for their polycistronic nature, small size and high “cleavage” efficiency. However, broad application of 2 A peptides is limited by the lack of systematic comparison of different 2As alone or in combination. Here we characterized the effect of varying gene position and 2As on the expression of proteins encoded in bi-, tri-, or quad-cistronic constructs. Using direct cardiac reprogramming as an example, we further determined the effect of varied 2As on the efficiency of fluorescent cell labeling and cell fate conversion. We found that the expression of fluorophores decreased as it was moved towards the end of the construct while reprogramming was most efficient with the fluorophore at the second position. Moreover, quad-cistronic TPE2A constructs resulted in more efficient reprogramming than 3P2A or PTE2A constructs. We expect that the bi-, tri-, and quad-cistronic vectors constructed here and our results on protein expression ratios from different 2A constructs could serve to guide future utilization of 2A peptides in basic research and clinical applications.

## Introduction

**Figure 1:**
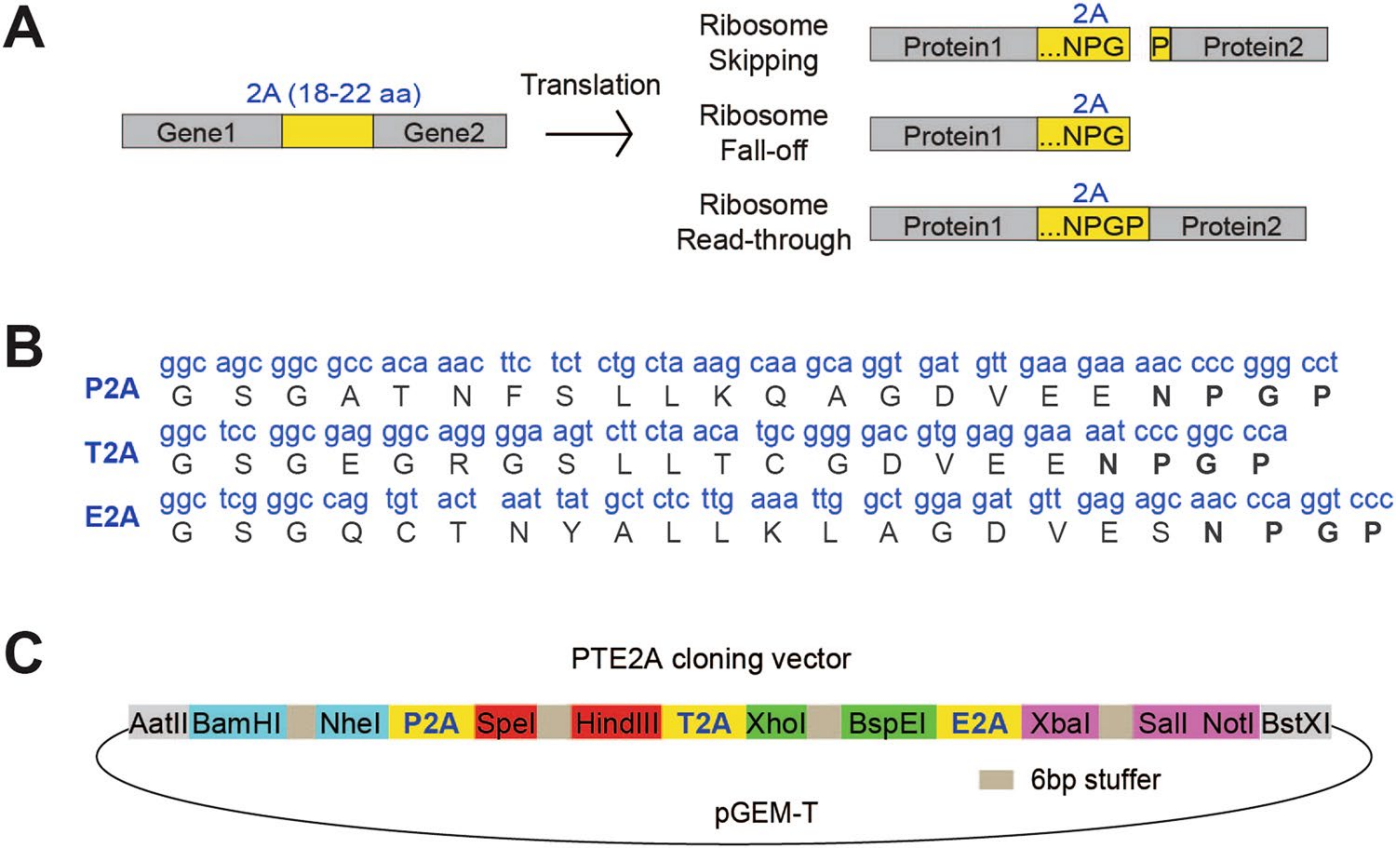
Construction of the multi-gene co-expression vector with 2A sequences. (**A**) Schematic representation of the mechanism of “self-cleaving” 2A peptides. (**B**) DNA and amino acid sequences of the various 2As used. A GSG linker was added to the N-terminus of all 2As. (**C**) A simplified map of the pGEM-T-PTE2A cloning vector showing 2As and restriction sites used.

Co-expression of multiple genes at a desired ratio is highly attractive for a broad array of basic research and biomedical applications including cellular reprogramming^1–5^, expression of multiple subunits of complex multimeric proteins in gene therapy^6–8^, tagging of protein of interest for live cell imaging or cell sorting^9–11^, and generation of efficient tools for fate mapping and genome editing^12–16^. Strategies for multigene co-expression include introduction of multiple vectors, use of multiple promoters in a single vector, fusion proteins, proteolytic cleavage sites between genes, internal ribosome entry sites, and “self-cleaving” 2A peptides. 2A peptides are 18–22 amino-acid (aa)-long viral oligopeptides that mediate “cleavage” of polypeptides during translation in eukaryotic cells^10, 17^. The designation “2A” refers to a specific region of the viral genome and different viral 2As have generally been named after the virus they were derived from. The first discovered 2A was F2A (foot-and-mouth disease virus)^18^, after which E2A (equine rhinitis A virus), P2A (porcine teschovirus-1 2A), and T2A (thosea asigna virus 2A) were also identified^19^. The mechanism of 2A-mediated “self-cleavage” was recently discovered to be ribosome skipping the formation of a glycyl-prolyl peptide bond at the C-terminus of the 2A^20, 21^ (Fig. 1A). A highly conserved sequence GDVEXNPGP is shared by different 2As at the C-terminus (Fig. 1B), and is essential for the creation of steric hindrance and ribosome skipping. There are three possibilities for a 2A-mediated skipping event (Fig. 1A). (1) Successful skipping and recommencement of translation results in two “cleaved” proteins: the protein upstream of the 2A is attached to the complete 2A peptide except for the C-terminal proline, and the protein downstream of the 2A is attached to one proline at the N-terminus. (2) Successful skipping but ribosome fall-off and discontinued translation results in only the protein upstream of 2A. (3) Unsuccessful skipping and continued translation resulting in a fusion protein. Overall, 2A peptides lead to relatively high levels of downstream protein expression compared to other strategies for multi-gene co-expression, and they are small in size thus bearing a lower risk of interfering with the function of co-expressed genes. 2A peptides have also been successfully employed by several different groups for polycistronic and bi-cistronic multigene expression^2, 6, 22–26^.

Despite the advantages of 2A peptides, widespread adoption has been limited by the lack of systematic comparisons between the different 2A sequences alone or in combinations. Previous studies primarily focused on the “cleavage” efficiencies of different 2As in a bi-cistronic setting and obtained mixed results, with some studies suggesting T2A while others showing P2A to have the highest efficiency^6, 20, 27^. In addition, the ratio of protein expression in a polycistronic construct and how it is affected by the utilization of different 2As are not known. There also is no widely-adopted 2A-containing cloning vector for polycistronic gene expression. In order to guide future design of optimal polycistronic constructs with predictable protein expression, we aimed to characterize the effect of gene position on protein expression level in bi-, tri-, and quad-cistronic 2A constructs and to determine whether different 2As, alone or in combination, affect the level of expressed proteins. To facilitate rapid and convenient cloning in the current study and future applications, we built a set of cloning vectors that contain three 2A sequences and four pairs of restriction sites for insertion of up to four genes (PTE2A in Fig. 1C, TPE2A and 3P2A). For gene co-expression, it is usually favorable to use fluorescent proteins (FPs) or surface markers to track and/or sort cells that express the vector. Therefore, we cloned various FPs in our polycistronic 2A constructs to enable convenient measurement of protein expression levels and to create templates for future applications. In summary, our study demonstrated the importance of 2A selection in achieving a desired ratio of protein expression and established the optimal 2A construct for co-expression of two, three, and four genes.

## Results and Discussion

### Bi-cistronic constructs: dramatically decreased protein expression at the second position and highest efficiency of expression obtained with T2A and tandem P2A-T2A (tPT2A)

**Figure 2:**
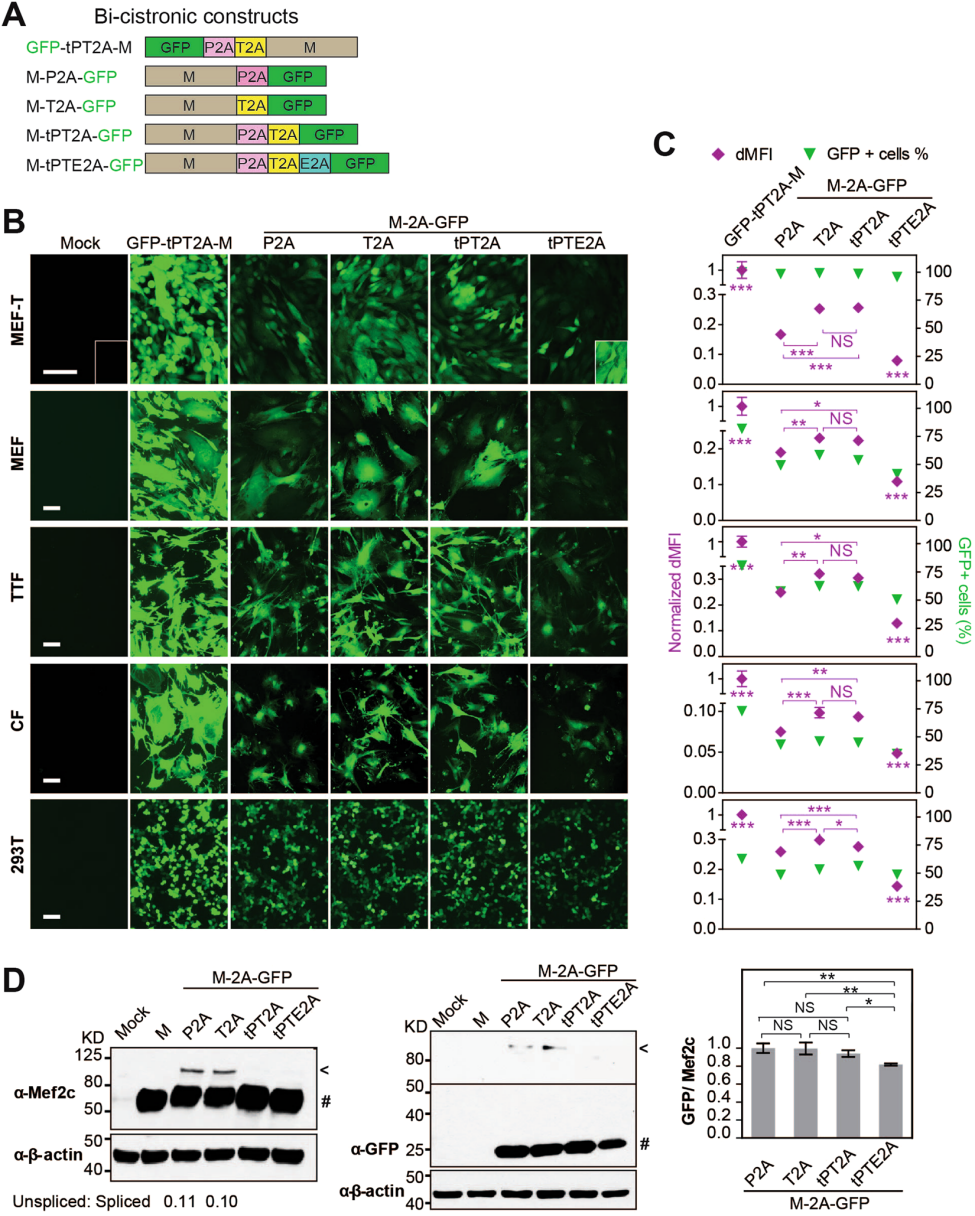
Bi-cistronic 2A constructs: impact of 2A sequences on the expression of the gene at the second position. (**A**) Different bi-cistronic 2A constructs encoding *Mef2c* (M) and *Gfp*. “t” is the abbreviation for “tandem.” (**B**) Representative live fluorescent images of various cell types transduced (except transfection in 293 T) with retroviruses (pMXs) encoding the different 2A constructs. MEF, mouse embryonic fibroblast. MEF-T, immortalized MEF cell line^22^. CF, primary cardiac fibroblast. TTF, primary tail-tip fibroblast. Boxed area in MEF-T Mock and M-tPTE2A-GFP are longer exposed to show that the percentage of GFP+ cells were 100% for M-tPTE2A-GFP even though GFP intensity is low. Images were taken on day 3 post-transduction or 48 hr post-transfection (293T) at 20X (40X for MEF-T). Scale bar = 100 µm. (**C**) Cells in (**B**) were collected and quantified for percentage of GFP+ cells (green triangle) and the fluorescence intensity of GFP (purple diamond) by flow cytometry. dMFI, delta median fluorescence intensity = MFI of GFP+ cells − MFI of GFP− cells. dMFI of all 2A constructs were normalized to that of the GFP-tPT2A-M. (**D**) Western blots showing Mef2c and GFP protein expression in MEF-T cells transduced with different bi-cistronic constructs. ^**#**^Spliced protein. ^<^Unspliced M-2A-GFP. Middle panel: a longer exposure was used to visualize the unspliced GFP. Right panel: the expression levels of unspliced Mef2c and GFP were quantified and normalized to the β-actin loading control and then GFP/Mef2c ratios in different M-2A-GFP constructs were calculated and further normalized to that in the M-P2A-GFP construct. Mean ± SD of triplicate experiments were shown. Statistical significance was calculated by one-way ANOVA and bonferroni correction.

To determine the ratio of protein expression between the first and the second gene positions in a bi-cistronic vector and to characterize the effect of a given 2A sequence on protein expression at the second gene position, we constructed a set of bi-cistronic vectors containing different 2As between *Mef2c* (M) and *Gfp* (Fig. 2A). In addition to P2A and T2A, which have both been reported to have the highest “cleavage” efficiency^6, 20, 27^, we also tested tandem 2As for bi-cistronic expression to determine whether tandem 2As might improve protein expression over individual 2As. *Gfp* was cloned downstream of 2As at the second gene position to serve as a reporter to determine each 2A’s efficiency. As a control for protein expression level at the first gene position, GFP-tPT2A-M (tandem P2A-T2A) was also constructed. These bi-cistronic 2A constructs were used to transfect human 293T cells. We also generated retroviruses expressing these bi-cistronic transgenes to enable transduction of multiple cell lines including mouse embryonic fibroblasts (MEF), MEF cell line transformed with large T antigen (MEF-T)^22^, neonatal mouse cardiac fibroblasts (CF) and tail-tip fibroblasts (TTF). Fluorescent images of these cells were taken at day 3 post-transfection/transduction (Fig. 2B), and the percentage of GFP+ cells and GFP fluorescence intensity in GFP+ cells were determined by flow cytometry (Fig. 2C). Comparison of GFP intensity from the GFP-tPT2A-M construct and all other constructs revealed that the GFP fluorescence at the second gene position was about 5–30% of that observed with GFP at the first gene position, depending on the cell type and 2A sequences used (Fig. 2C, purple diamond). Comparison of GFP intensity among the different 2A bi-cistronic constructs demonstrated that protein expression at the second gene position was equivalently greatest in the T2A and tPT2A constructs, slightly less in the P2A construct, and lowest in the tPTE2A (tandem P2A-T2A-E2A) construct (Fig. 2B and C). This finding was consistent across all tested cells and in accordance with previous reports that T2A resulted in the least amount of “uncleaved” protein product among different 2As^6, 20^. Similar results on GFP intensity were observed with the percentage of GFP+ cells (green triangle, Fig. 2C). Furthermore, we also used western blotting to determine the efficieny of 2A-mediated “cleavage” and the protein expression level of GFP (Fig. 2D). No uncleaved band was observed for tandem constructs tPT2A or tPTE2A, suggesting complete cleavage by tandem 2As. A very faint uncleaved band was observed for single 2A constructs P2A and T2A on both Mef2c and GFP blots. Quantification showed that the ratio of uncleaved to cleaved Mef2c is ~0.1 in both P2A and T2A constructs. Further quantification demonstrated that the ratio of expression of cleaved GFP to cleaved Mef2c was equivalently high in P2A, T2A, and tPT2A constructs, which is about 20% higher than observed in tPTE2A constructs. The slight difference in GFP expression between flow cytometry and western blotting for the P2A construct may be due to the relatively lower sensitivity of western blotting compared to FACS and/or the semi-quantitative nature of western blotting. These flow cytometry and western blotting results also suggest that the ratio of occurrence of ribosome skipping and recommencement of translation: ribosome skipping and fall-off and discontinued translation: ribosome read-through is about 30% to 60% to 10%, which is consistent with previous findings using F2A that there was a molar excess of ‘cleavage’ product N-terminal of 2A over the product C-terminal of 2A^21^. Taken together, our results here suggest that protein expression decreases by ~70% at the second gene position compared to the first gene position in bi-cistronic 2A constructs, tPT2A leads to no uncleaved protein product, and both tPT2A and T2A lead equivalently to the highest level of protein expression at the second gene position compared to other 2As.

### Tri-cistronic constructs: highest protein expression at the first position and lowest at the second position

**Figure 3:**
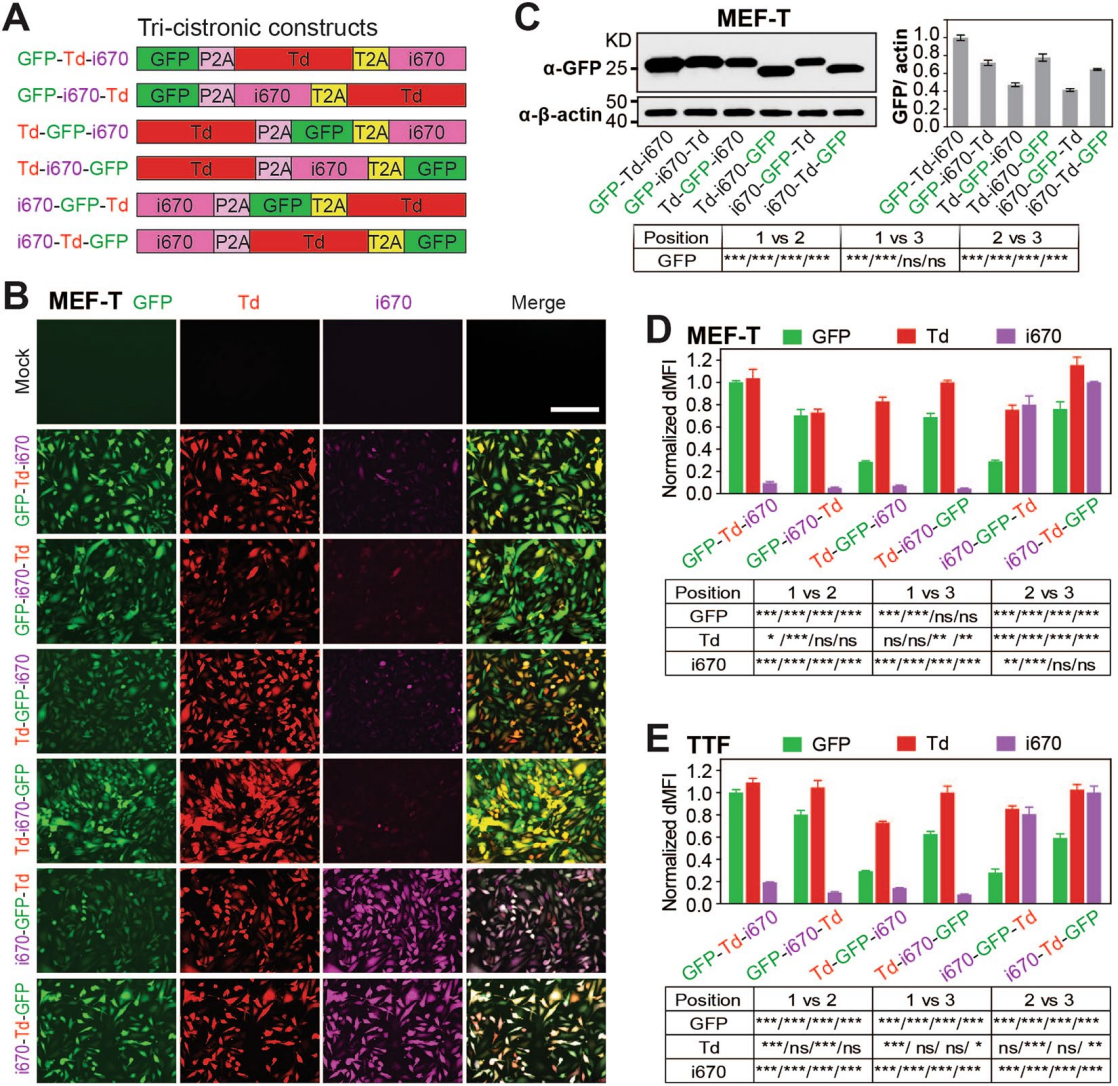
Tri-cistronic 2A constructs: positional effects on gene expression. (**A**) Tri-cistronic constructs in a P2A-T2A backbone expressing three FPs GFP, Td and i670 in different orders. (**B** and **C**) MEF-T cells were transduced with retroviruses (pMXs) encoding the different 2A constructs for 3 days. Live fluorescent images of these cells were taken at 20X (**B**) and then cells were collected for (**C**) western blotting analysis for GFP expression, or (**D**) flow cytometry analysis for dMFI of GFP (green bar), Td (red bar), and i670 (purple bar). (**C**) GFP was quantified and normalized to the β-actin loading control. (**D**) dMFI was normalized to that of GFP-Td-i670 for GFP, Td-i670-GFP for Td, and i670-Td-GFP for i670. (**E**) The same as (**D**) except performed in TTF. Mean ± SEM of multiple experiments were shown. Results from one-way ANOVA and bonferroni correction were summarized in a table. Scale bar = 200 µm.

To determine relative protein expression at different positions along a tri-cistronic construct, we generated a complete set of six vectors containing fluorophores *Gfp*, *Tdtomato* (Td), and far red fluorscent protein *iRFP670* (i670) varied in all possible positions with intervening P2A and T2A sequences (Fig. 3A). The three selected FPs represent a wide range of brightness, with Td as the brightest FP available whose brightness triples that of GFP^28^, and i670 as a relatively dim FP whose brightness is one third of GFP’s^29^. Live fluorescent images of GFP, Td, and i670 were captured in MEF-T cells at 3 days post-transduction (Fig. 3B) and MEF-T cells were collected and analyzed by western blotting for GFP expression (Fig. 3C) and by flow cytometry for fluorescence intensity of all three FPs (Fig. 3D). With both western blotting and flow cytometry, comparison of the expression of GFP at various positions in tri-cistronic constructs revealed that their protein levels were highest at the first position, less at the third position, and lowest at the second position. The same trend was observed by flow cytometry for the expression of i670. Theoretically a gradual decrease of translated protein along a polycistronic vector is expected due to naturally occurring ribosome drop-off at a constant rate that is positively correlated with transcript length^30^. Yet it is possible that the higher level of protein expression at the third gene position over the second position resulted from the use of T2A immediately upstream of the third gene, given that T2A led to higher expression of the downstream protein than did P2A in bi-cistronic constructs (Fig. 2C). More interestingly, positional effects on the level of expressed protein seems to be negatively correlated with the brightness of the FP. The brightest FP, Td, did not exhibit dramatic changes in fluorescence intensity when moved through different positions in the tri-cistronic construct, yielding an intensity range of 73%–116% when compared to the intensity at the first position (red bar, Fig. 3C). The less bright GFP led to ~30% decrease in intensity at the third position and ~70% decrease at the second position when compared to its intensity at the first position (green bar, Fig. 3C), and the least bright i670 resulted in ~90% and ~95% decrease in intensity at the third and second positions, respectively, when compared to its intensity at the first position (purple bar, Fig. 3C). Flow cytometry analysis was also performed on TTFs transduced with all tri-cistronic constructs (Fig. 3E) and the data were consistent with results obtained in MEF-T (Fig. 3D). Protein expression levels and functionality from a polycistronic 2A construct could be potentially affected by the N- or C-terminal fusion of partial 2A sequences, and/or the identity and order (context) of adjacent genes^31^. Therefore, care must be taken when applying conclusions from previous 2A studies or the current one to other genes or experimental contexts. Taken together, the different levels of protein expression observed at varied positions along our tri-cistronic constructs likely reflect the combined effects of position along the construct, efficiency of protein expression mediated by different 2A sequences, and the brightness of the FPs.

### Quad-cistronic constructs: protein expression gradually decreases toward the 3′ end and TPE2A-MTdGT was the optimal construct for cell tracking and reprogramming

**Figure 4:**
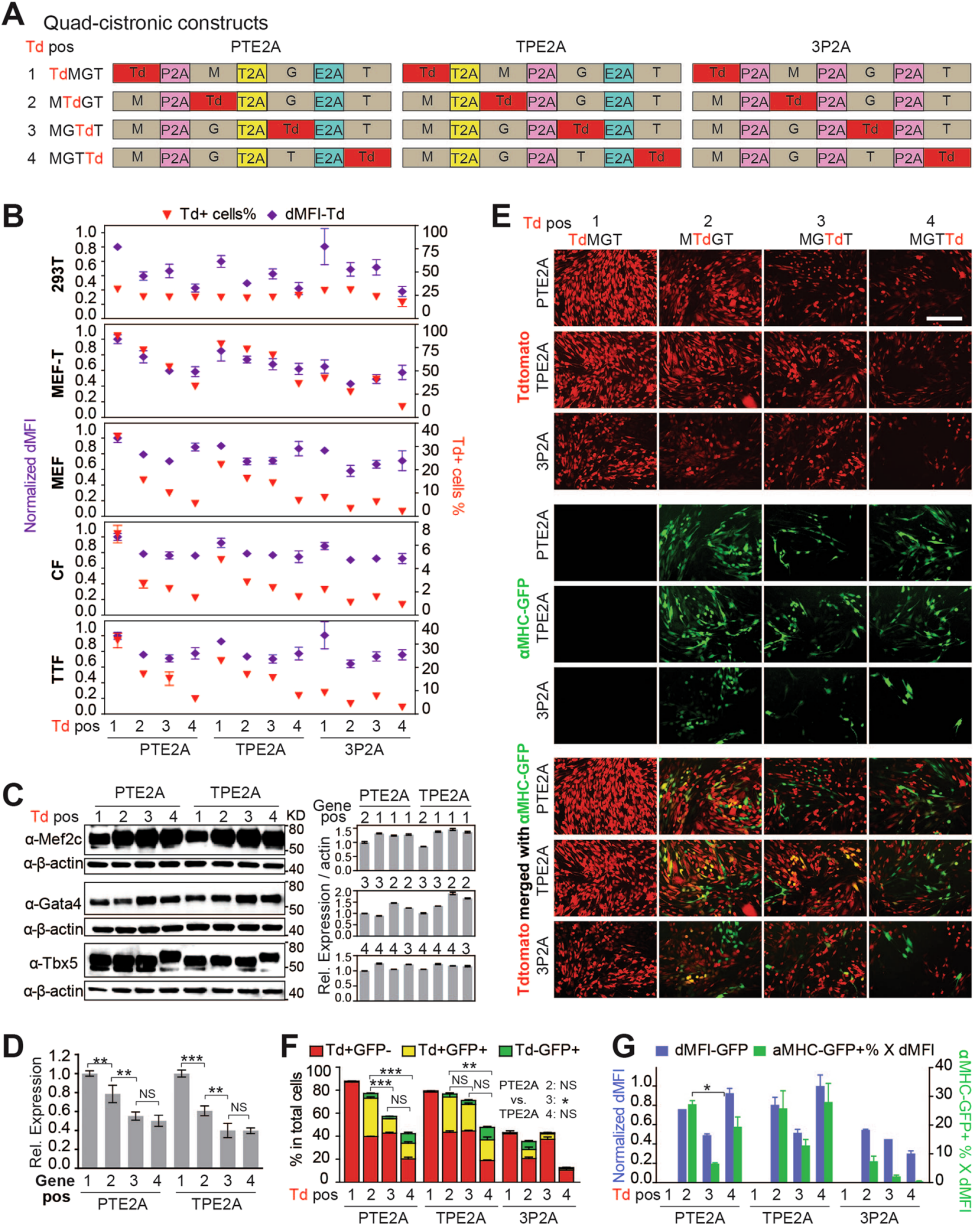
Quad-cistronic 2A constructs: example of application in cellular reprogramming. (**A**) Quad-cistronic constructs using different orders of 2As (PTE2A, TPE2A, and 3P2A) for the expression of *Mef2c* (M), *Gata4* (G), *Tbx5* (T) and *Tdtomato* (Td). A total of 12 constructs were built with Td rotating from position 1 to 4. (**B**) Flow cytometry quantification of the percentage of Td+ cells (red triangle) and dMFI of Td (purple diamond) in various cell types transduced (except transfection in 293T) with retroviruses (pMXs) encoding the different 2A constructs. dMFI of Td was normalized to that in the PTE2A-TdMGT construct. Cells were collected on day 3 post-transduction or 48 hr post-transfection (293T). (**C**) Western blots for M, G, T expression in PTE2A and TPE2A constructs. Right panel: quantification after normalization to the β-actin loading control. (**D**) Positional effects on protein expression in quad-cistronic constructs calculated based on (**C**). (**E–G**) Td expression and reprogramming with different 2A constructs in MEF-T on day 3 post-transduction. (**E**) Representative live fluorescent images of Td and the reprogramming reporter αMHC-GFP. All taken at 20X. Scale bar = 200 µm. (**F**) Flow cytometry quantification of Td and GFP single- and double-positive cells. (**G**) GFP dMFI in αMHC-GFP+ cells (blue bar) and the product of the percentage of αMHC-GFP+ cells and dMFI-GFP (green bar). dMFI of GFP was normalized to that of the TPE2A-MGTTd construct. Mean ± SD of triplicate experiments were shown in (**B**,**D**,**E**).

Co-expression of more than three genes is sometimes desired, e.g., for studies of induced pluoripotency with the Yamanaka four factors^32^. Here we rotated the position of Td from the first to fourth position in a quad-cistronic construct in order to characterize its protein expression level at each position. We also utilized the M, G (*Gata4*), and T (*Tbx5*)-induced cardiac reprogramming system as an example to demonstrate how to identify a 2A vector optimal for tracking reprogramming factor uptake and expression, as well as reprogramming outcomes. We decided to fix the relative positions of the reprogramming factors as M upstream of G, and G upstream of T, according to our recent findings that this order leads to optimal reprogramming in a tri-cistronic construct^33^. We cloned three sets of vectors with different combinations of 2As (Fig. 4A). The first two sets, PTE2A and TPE2A, include one each of the three most efficient 2As available, but with different orders of P2A and T2A, based on the findings from our bi-cistronic constructs. The last set consists of three P2As to test whether multiple identical 2A sequences with exactly the same DNA sequences can be used, which has not previously been tested in the literature.

We first determined the expression of all four genes after introducing quad-cistronic 2A constructs into various cell lines (293T transfection and MEF-T transduction) and primary cells (MEF, CF, TTF transduction). Td expression was quantified by flow cytometry (Fig. 4B) and M, G, T expression was determined by western blotting (Fig. 4C and D). Comparison of the percentage of Td+ cells (red triangle) across all 12 constructs revealed a gradual decrease of Td+ cells% from the 5′ to 3′ end of the construct in all three vector sets, with much lower Td+ cells% from 3P2A vectors at each Td position than from PTE2A and TPE2A vectors (except for transfection of 293T). These data suggest that protein expression in a relatively long (6 kilobase) polycistronic construct is mostly affected by protein position rather than by 2A sequences and their order, possibly due to a more prominent effect of ribosome drop-off in longer constructs. These data also imply that multiple identical 2As should not be used in the same construct, which leads to hampered protein expression. Similar to tri-cistronic constructs, Td fluorescence intensity (purple diamond) from quad-cistronic constructs in most cell types did not change at position 2, 3, and 4, even though about a 20–30% reduction was observed when comparing Td intensity at position 2–4 to that observed at the first position. Additionally, MEF-T cells, where transduction efficiency was the highest (80–100%) among all cells, demonstrated gradually decreasing Td intensity as Td’s position was shifted toward the 3’ end, with the exception of Td intensity at positions 3 and 4, which was very similar in PTE2A and TPE2A constructs (Fig. 4B). Consistent with Td intensity, quantification of western blots of M, G, T expression in MEF-T cells also demonstrated a gradual decrease in protein expression from position 1 to 2, and from position 2 to 3, but similar expression levels at position 3 and 4 (Fig. 4C and D). These data suggest that both gene position and transduction efficiency affect protein expression in a long quad-cistronic construct.

Next, we performed direct cardiac reprogramming in MEF-T cells with all 12 constructs. These MEF-T cells harbor a transgenic GFP reporter under the control of the αMHC promoter and turn on GFP expression upon reprogramming^22^. Fluorescent images of both Td and GFP were taken at day 3 post-transduction (Fig. 4E), and the percentage of FP+ cells and FP intensities were determined by flow cytometry (Fig. 4E and F). No GFP+ cells were detected when Td was at the first position, consistent with our previous finding that high M expression at the 5′ end of the construct leads to optimal reprogramming^33^. On the other hand, GFP+ cells were observed with the introduction of all other constructs, in which Td was at position 2, 3, or 4. The most GFP+ (yellow combined with green bars, Fig. 4F) and Td+ GFP+ cells (yellow bars, Fig. 4F) were detected from PTE2A and TPE2A constructs with Td at the second position. In general, PTE2A and TPE2A constructs led to more GFP+ cells than 3P2A constructs. TPE2A constructs demonstrated similar reprogramming efficiency to PTE2A constructs with Td at position 2 and 4, but showed more GFP+ cells and more Td+ GFP+ cells than the PTE2A construct when Td was at the third position. When Td was at the last position in PTE2A and TPE2A constructs, even though a similar number of GFP+ cells was observed compared to when Td was at the second position in these constructs, many of these cells were negative for Td. Comparison of GFP intensity across these different 2A constructs revealed that GFP intensity was highest in PTE2A and TPE2A constructs with Td at position 2 and 4 (Fig. 4G), which was consistent with our conclusions based on the percentage of GFP+ cells (Fig. 4F). Taken together, the TPE2A construct with Td at the second position (MTdGT) led to balanced expression of Td and reprogramming factors, and is considered optimal for tracking cardiac reprogramming in MEF-T cells.

In summary, our results in this study provide useful information that may guide thoughtful design of bi-, tri-, and quad-cistronic constructs that yield desired ratios of gene co-expression. We expect that our results may serve as a reference table or initial framework for designing polycistronic vectors, which may help in achieving the goals of individual studies.

## Methods

### Mouse lines and cells

All animal experiments conformed to the NIH guidelines (Guide for the care and use of laboratory animals) and UNC Qian Lab animal protocol #15.277.0. This protocol was approved by The University of North Carolina at Chapel Hill Institutional Animal Care and Use Committee (IACUC) that oversees the University’s animal care and use (NIH/PHS Animal Welfare Assurance Number: A3410-0; USDA Animal Research Facility Registration Number: 55-R-0004; AAALAC Institutional Number: #329). Transgenic mice from CD1 background that harbor αMHC-GFP reporter^3, 33, 34^ were maintained in accordance with guidelines established in our IACUC protocol. They were used for the isolation of MEF (E13.5), primary neonatal (day 1.5) explant CF and neonatal TTF as previously described^35^. MEF-T is a cell line selected from SV40 large-T antigen-transformed MEF and it was generated recently by our lab as described^22^. MEF (passage 3–10) and MEF-T were maintained in growth media: DMEM containing 10% Fetal Bovine Serum (FBS) and 50 μg ml^−1^ penicillin/streptomycin (Sigma). CF and TTF were maintained in fibroblast media (IMDM/20% FBS).

### Plasmids

Cloning of all constructs was performed using the pGEM-T easy vector (Promega) as an intermediate. First, a 300 base pair (bp) DNA fragment containing desired restriction endonuclease sites and 2A-encoding sequences were synthesized (Genewiz) and the sequences were listed in Table S1. A 6 bp stuffer sequence was placed in between each pair of endonuclease sites for gene insertion. Then sequences between AatII and BstXI on pGEMT-T were replaced with the synthesized DNA fragment PTE2A or 3P2A to obtain the cloning intermediates pGEM-T-PTE2A and pGEMT-T-3P2A. Next, genes of interest were PCR-amplified and inserted into the cloning intermediates one by one. The templates used for PCR are: pMXs-MGT^33^ for M, G, T, pLenti-GFP (Cell Biolabs, LTV-400) for GFP, pCSCMV-tdTomato (Addgene) for Td, and piRFP670-N1 (Addgene, #45457) for iRFP670. For quad-cistronic constructs, the restriction sites used for each gene position were: first, BamHI and NheI, second, SpeI and HindIII, third, XhoI and BspEI, last, XbaI and SalI. All bi-cistronic constructs were cloned in the intermediate plasmid pGEM-T-PTE2A, and the restriction sites used depended on the 2A sites in between the first and the second gene. For M-T2A-GFP, BamHI and HindIII were used for the insertion of M, and XhoI and SalI were used for the insertion of GFP. For all other bi-cistronic constructs in which P2A immediately followed the first gene, BamHI and NheI were used for the first gene, and the restriction sites used for the second gene vary by 2A sites: SpeI and SalI for M-P2A-GFP, XhoI and SalI for M-tPT2A-GFP and GFP-tPT2A-M, and XbaI and SalI for M-tPTE2A-GFP. All tri-cistronic constructs were also cloned in the intermediate plasmid pGEM-T-PTE2A, and the restriction sites used were: BamHI and NheI for the first gene, SpeI and HindIII for the second gene, and XhoI and SalI for the third (last) gene. A 6 bp kozak sequence ACCGCC was added right before the ATG start codon of the first gene in every construct and the stop codon TAA was added at the end of the last gene in every construct. Finally, the constructs were excised from pGEM-T and inserted into the pMXs retroviral vector (Cell Biolabs) with BamHI and SalI. To construct the quad-cistronic TPE2A constructs, P2A and T2A were PCR amplified and swapped based on PTE2A constructs.

### Retrovirus packaging and transduction

293T cells stably expressing packaging plasmids (PlatE, Cell Biolabs) were maintained in growth media supplemented with 1 μg ml^−1^ puromycin (Sigma), and 100 μg ml^−1^ of blasticidin S (Life Technologies). One day before transfection, 4–5 × 10^6^ cells were seeded onto 10 cm dish in growth media without puromycin and blasticidin. The next day, pMXs-based retroviral vectors were introduced into PlatE cells using Nanofect (ALSTEM). Generally, 20 μg of plasmid DNA was combined with 500 μl plain DMEM and 45 μl of Nanofect reagent was combined with 500 μl plain DMEM. The Nanofect suspension was added into the DNA suspension and the mixture was vortexed for 15 seconds, kept at room temperature for 15 minutes, and then added dropwise to the PlatE cells. Culture media were changed to fresh media right before transfection and all reagents used were warmed to room temperature before mixing. Transfected cells were then incubated overnight at 37 °C with 5% CO_2_. Medium was changed the next day and virus-containing supernatant was collected 48 hours after transfection, filtered through a 0.45 μm cellulose acetate filter (Thermo Scientific) and incubated with PEG8000 (Sigma, 4 volumes of supernatant and 1 volume of 40% PEG8000/PBS) overnight at 4 °C. Target cells were plated the same day as virus collection and precipitation at a density of 1 × 10^4^ cm^−2^ on 0.1% gelatin-coated 24 or 12 well-plate. On the second day, viruses were pelleted by centrifuge at 4000 x*g*, 4 °C for 30 minutes, re-suspended with growth media or fibroblast media supplemented with 4 μg ml^−1^ polybrene (Life Technologies), and added to target cells immediately. Twenty-four to 48 hours after infection, the virus-containing medium was replaced with with growth media or fibroblast media.

### Immunofluorescence imaging and flow cytometry

At day 3 post-transduction, cells were washed three times with PBS and images of live cells were acquired using EVOS® FL Auto Cell Imaging System (Life Technologies). For flow cytometry, cells were first dissociated with 0.05% trypsin/EDTA (Life Technologies) for 5 minutes at 37 °C and then trypsin was neutralized with growth media. The cells were then pelleted, resuspended with FACS buffer (PBS supplemented with 2% FBS and 2 mM EDTA), and analyzed by BD Accuri™ C6 flow cytometer or LSRII (BD Bioscience).

### Western Blot

Cells were collected and lysed with RIPA buffer supplemented with protease inhibitor and phenylmethanesulfonyl fluoride (PMSF) for 15 minutes on ice. The lysate was then centrifuged at 16000 x*g* for 10 minutes and supernatant was added with 4x SDS loading buffer (Bio-Rad) and boiled for 5 minutes at 95 °C. Cleared lysate was run on a 4–15% gradient SDS-PAGE gel (Bio-Rad) and proteins were transferred to nitrocellulose membranes. After blocking with 5% milk, proteins were probed with primary antibodies: rabbit anti-GFP (Invitrogen, 1:500), rabbit anti-Mef2c (Abcam, 1:1000), goat anti-Gata4 (Santa Cruz Biotechnology 1:200), and goat anti-Tbx5 (Santa Cruz Biotechnology, 1:200). The target proteins were detected by chemiluminescence (ECL, Thermo Scientific). The membranes were then stripped with stripping buffer (Sigma) for 12 minutes and re-probed with mouse anti-β-actin (Santa Cruz Biotechnology, 1:1000) as the loading control. Quantification was performed with Image J.

### Statistical analyses

Where appropriate, values are expressed as the mean ± standard deviation (SD) or standard error of mean (SEM) of at least triplicate experiments. Statistical analyses were performed with one-way analyses of variance (ANOVA) followed by Bonferroni correction. A P value of <0.05 was considered statistically significant (*), a P value of <0.01 was considered highly significant (**), and a P value of <0.001 was considered strongly significant (***). All data are representative of multiple repeated experiments.

## Electronic supplementary material

Supplementary Informaiton
