# Supplementary material for: Systematic comparison of 2A peptides for cloning multi-genes in a polycistronic vector
Source: Sci Rep. 2017 May 19;7:2193. doi: 10.1038/s41598-017-02460-2 (PMC5438344; doi:10.1038/s41598-017-02460-2)
Supplement: Supplementary file 1 — Supplementary Informaiton [file 41598_2017_2460_MOESM1_ESM.pdf]

Supplementary information for:

Systematic comparison of 2A peptides for cloning multi-genes in a polycistronic vector

Ziqing Liu[1,2,3], Olivia Chen[1,2,3], J. Blake Joseph Wall[1,2,3], Michael Zheng[1,2,3], Yang Zhou[1,2,3], Li Wang[1,2,3], Haley Ruth Vaseghi[1,2,3], Li Qian[1,2,3,*], and Jiandong Liu[1,2,3,*]

[1]Department of Pathology and Laboratory Medicine, [2]McAllister Heart Institute, [3]Lineberger Comprehensive Cancer Center, University of North Carolina, Chapel Hill, NC 27599.

Table S1 Sequences of quad-cistronic vectors.

PTE2A backbone

AatII BamH I ovehang1 Nhe I P2A SpeI overhang2 HindIII T2A XhoI overhang3 BspEI E2A XbaI overhang4 Sal I NotI BstXI

GACGTC GGATCC GCT GGA GCTAGC ggc agc ggc gcc aca aac ttc tct ctg cta aag caa gca ggt gat gtt gaa gaa aac ccc ggg cct ACTAGT GCTGGA AAGCTT ggc tcc ggc gag ggc agg gga agt ctt cta aca tgc ggg gac gtg gag gaa aat ccc ggc cca CTCGAG GGAGCA TCCGGA ggc tcg ggc cag tgt act aat tat gct ctc ttg aaa ttg gct gga gat gtt gag agc aac cca ggt ccc TCTAGA GGAGCA GTCGAC GCGGCCGC CCAACGCGTTGG

TPE2A backbone

AatII BamH I ovehang1 Nhe I T2A SpeI overhang2 HindIII P2A XhoI overhang3 BspEI E2A XbaI overhang4 Sal I NotI BstXI
GACGTC GGATCC GCT GGA GCTAGC ggc tcc ggc gag ggc agg gga agt ctt cta aca tgc ggg gac gtg gag gaa aat ccc ggc cca ACTAGT GCTGGA AAGCTT ggc agc ggc gcc aca aac ttc tct ctg cta aag caa gca ggt gat gtt gaa gaa aac ccc ggg cct CTCGAG GGAGCA TCCGGA ggc tcg ggc cag tgt act aat tat gct ctc ttg aaa ttg gct gga gat gtt gag agc aac cca ggt ccc TCTAGA GGAGCA GTCGAC GCGGCCGC CCAACGCGTTGG

3P2A backbone

AatII BamH I ovehang1 Nhe I P2A SpeI overhang2 HindIII P2A XhoI overhang3 BspEI P2A XbaI overhang4 Sal I NotI BstXI

GACGTC GGATCC GCT GGA GCTAGC ggc agc ggc gcc aca aac ttc tct ctg cta aag caa gca ggt gat gtt gaa gaa aac ccc ggg cct ACTAGT GCTGGA AAGCTT ggc agc ggc gcc aca aac ttc tct ctg cta aag caa gca ggt gat gtt gaa gaa aac ccc ggg cct CTCGAG GGAGCA TCCGGA ggc agc ggc gcc aca aac ttc tct ctg cta aag caa gca ggt gat gtt gaa gaa aac ccc ggg cct TCTAGA GGAGCA GTCGAC GCGGCCGC CCAACGCGTTGG
